# Factors influencing canine rabies vaccination among dog-owning households in Nigeria

**DOI:** 10.1016/j.onehlt.2024.100751

**Published:** 2024-05-10

**Authors:** Philip P. Mshelbwala, Charles E. Rupprecht, Modupe O. Osinubi, Emmanuel O. Njoga, Terese G. Orum, J. Scott Weese, Nicholas J. Clark

**Affiliations:** a Faculty of Veterinary Medicine, University of Abuja, Nigeria; b Department of Primary Industries, NSW, Australia; c College of Forestry, Wildlife & Environment, College of Veterinary Medicine, Auburn University, Auburn, AL, USA; d Centers for Disease Control and Prevention, Atlanta, GA, USA; e Department of Veterinary Public Health and Preventive Medicine, Faculty of Veterinary Medicine, University of Nigeria, Nigeria; f Regional Disease Surveillance System Enhancement Project, Abuja, Nigeria; g Department of Pathobiology, Ontario Veterinary College, Guelph, Canada; h School of Veterinary Science, The University of Queensland, Australia

**Keywords:** Nigeria, Rabies, Bayesian, Dog, Vaccination, Risk factors, Modelling

## Abstract

Rabies perpetuates in Nigeria despite initiatives like the Regional Disease Surveillance System Enhancement Project, with evidence indicating suboptimal canine vaccination rates as a contributing factor. To inform effective planning of mass dog vaccination campaigns, it is crucial to understand the factors associated with variation in canine vaccination rates. We conducted a cross-sectional study in 2022 to understand factors associated with canine vaccination. We used stratified random sampling of the streets and dog-owning households to survey 4162 households from three states and the Federal Capital Territory (FCT). We then built a joint probabilistic model to understand factors associated with dog vaccination and non-vaccination. First, we modelled rabies knowledge as a latent variable indirectly measured with several targeted survey questions. This method allowed a respondent's unobserved understanding of rabies to be estimated using their responses to a collection of survey questions that targeted different aspects of rabies epidemiology and took various possible response distributions (i.e., ordinal, categorical, binary). Second, we modelled factors influencing pet owners' decisions to vaccinate their dogs against rabies and barriers to dog vaccination among dog owners whose dogs were not vaccinated against rabies. Posterior distributions revealed that the probability of dog vaccination was positively associated with the owner's latent knowledge of rabies, civil servant service employment, residence in the FCT, ownership of a single dog, providing care to dogs, and a preference for contemporary treatment following a dog bite. Conversely, non-vaccination was positively associated with private employment, residing in Anambra and Enugu states, owning multiple dogs, allowing dogs to search for leftovers, and a preference for traditional treatment after a dog bite. Cost was the primary barrier against vaccination for dog owners in Anambra and Enugu, while mistrust posed a major challenge for those in the FCT. Owners in areas with veterinary establishments cited cost as a barrier, while those without a veterinary establishment cited access as the primary barrier. Our study underscores the need to enhance rabies knowledge, tailor vaccination campaigns to specific demographics, address financial and access barriers, and combat hesitancy to improve rabies vaccination rates in Nigeria.

## 1 Introduction

Annually, tens of thousands of people die from rabies in low- and middle-income countries (LMICs) [1,2]. Most of these deaths are acquired through the percutaneous bite of rabies virus-infected dogs [3]. This zoonosis causes approximately 3.7 million disability-adjusted life years and 8.6 billion US dollars in economic losses each year [1]. Canine rabies has been eliminated in all highly developed countries with a Human Development Index (HDI) > 0.9 [4]. However, most LMICs, particularly throughout sub-Saharan Africa, still grapple with this challenge [4,5]. Previous studies showed that to attain the appropriate herd immunity to break the transmission of dog-mediated rabies, up to 70% of susceptible dogs in a specific area must be vaccinated against rabies annually [[6], [7], [8], [9]]. While there are multiple initiatives towards rabies prevention and control in sub-Saharan Africa by international and local authorities, no country in the region is free of canine rabies [4,5].

Nigeria is the most populous country in Africa, with a human population of over 200 million [10]. Although likely present historically, rabies was only reported in Nigeria in humans during 1912 [11]. After that, there have been notable outbreaks of human and animal rabies throughout the country [11,12]. General rabies knowledge varies, with children residing within academic institutions and attending schools being more aware of rabies than those outside the campus and not enrolled in school [13]. Mass dog vaccination is the most effective strategy to eliminate dog-mediated human rabies deaths [2,14,15]. However, reports across Nigeria suggest that rabies vaccination is suboptimal despite ongoing outbreaks [12,16].

In a recent study conducted in the FCT, individuals who were bitten by unvaccinated dogs had a higher probability of rabies-related deaths [17]. Clearly, we need to understand why so many dogs remain unvaccinated in Nigeria. Determining what social, economic, or other barriers prevent dog owners from vaccinating their dogs against rabies is necessary to inform strategic interventions that aim to increase vaccination coverage and break the transmission of canine rabies [18].

Regression models are commonly used to quantify factors associated with dog vaccination [16,19]. Some factors associated with dog vaccination include owner-related descriptors, such as knowledge about rabies and the ability to handle the dog, as well as environmental descriptors, such as distance and topography to a vaccination point and cultural and spiritual determinants [[20], [21], [22], [23]]. These provide useful information, but we still do not know which factors are associated with dog vaccination and non-vaccination in Nigeria. Within the context of canine-mediated rabies, does variation in knowledge on rabies track cultural or social divides? How might variation in rabies knowledge influence decisions to vaccinate? Knowledge will always be a latent variable because we can never fully measure it. A causal diagram framework overcomes this challenge by allowing separate and joint models to quantify each exposure's total and direct effects on the outcome [24]. A Directed Acyclic Graph (DAG) has been used to guide variable selection for modelling risk factors for hospitalisation after a dog bite [25]. All factors evaluated were found to be associated with the risk of hospitalisation. The DAG is a graphical exploratory generative model that permits the derivation of a causal estimand [26,27]. Every research question subjected to the model might use a different statistical approach that includes manifest or latent variables [28].

This paper investigates the variation in rabies knowledge among Nigerian dog owners, explores dog ownership practices, and identifies sociodemographic factors associated with vaccination or non-vaccination of dogs using a joint probabilistic model. First, we model rabies knowledge (understanding) as a latent variable indirectly measured with several targeted survey questions. This method allowed a respondent's unobserved knowledge of rabies to be estimated using their responses to a collection of survey questions that target different aspects of rabies epidemiology and that took various possible response distributions (i.e., ordinal, categorical, binary). Similar approaches have been used in diverse fields to target questions related to latent knowledge or ability [29,30]. Secondly, we used a causal analysis of the factors associated with dog vaccination and the barriers to vaccination among a subset of dog owners whose dogs were not vaccinated against rabies. Findings from this study will help inform the effective planning of programs to improve Nigeria's current canine vaccination rate.

## 2 Materials and methods

### 2.1 Ethical considerations

The study received approval from the FCT Health Research Ethics Committee (FCT HREC) under the reference /FHREC/2019/01/04/21–01-19. Additionally, we obtained verbal consent from all participants included in the survey.

### 2.2 Study area

Nigeria is located within West Africa and comprises 36 states and the FCT. Nigeria borders the Republic of Niger in the North, Chad in the northwest, Cameroon in the east and Benin in the west. Nigeria is the seventh most populous country globally, with an estimated human population of 208,580,545 as of December 2020 (based on United Nations data) [10]. It is divided into 774 Local Government Areas (LGAs). The data used for this study were collected from three states (Anambra, Enugu, and Kaduna) and the FCT (Supplementary file 1).

### 2.3 Study design

#### 2.3.1 Criteria for study site selection

We conducted a cross-sectional study to understand the determinants of dog vaccination or non-vaccination in high-risk locations within Nigeria. We selected locations where data would be collected by stratification, using the level of rabies reporting by states from the national surveillance data and reports of rabies from published studies [31], which suggested more canine rabies in the Northwest [[32], [33], [34], [35]], Northcentral [[36], [37], [38], [39], [40]] and Southeast [[41], [42], [43], [44]]. We selected Kaduna in the Northwest (Ikara and Sabon Gari LGAs), FCT in the northcentral (Kuje and Bwari LGAs), and Enugu and Anambra (Idemili South and Ekwusigo) in the Southeast.

#### 2.3.2 Data collection

A team of 52 student volunteers was recruited and trained to survey between June and December 2022. Starting from the 1st major street in the selected area, we applied a systematic random sampling technique to survey every 5th street. Within each chosen street, sampling began from the 1st house on either side, selecting every 10th house for interviews with an adult resident [45,46]. In instances where this systematic approach could not be implemented due to the absence of clearly defined road networks, we either relied on informal directions provided by community members or utilised polio house markings as a guide to locate households with dogs [16,45]. In instances where we examined every tenth house and found no dogs, we proceeded to the next house in sequence. The same procedure applied to households where residents declined to participate in the interviews. We skipped houses without dogs and those that declined to participate for the next houses that possessed dogs and were willing to participate in the survey. A total of 261 houses declined to participate in the survey. The questionnaire was administered to an adult house member in English. To cater to respondents with limited English proficiency, translations were provided in the three primary languages commonly spoken in Nigeria: Hausa, Igbo, and Yoruba. These translations encompassed the names by which rabies is recognised throughout Nigeria, including digbolugi (Yoruba), ciwon kare (Hausa) and arankita (Igbo) [47]. The questions targeted three primary groups of information. First, sociodemographic characteristics of the dog owner, including age, gender, level of education (categorical), occupation (categorical), number of people (discrete), and dogs living in the household (discrete). For the occupation of the dog owner, the categories included students, civil servants, and those in the private sector (encompassing the unemployed and individuals running their own businesses). Second, we asked about dog-keeping practices such as the major use of the dog (categorical), whether the dog was under confinement, partial confinement or no confinement (categorical). Confined dogs were securely enclosed within designated areas, such as fenced yards or kennels, under the control of their owners. Partially confined dogs had limited boundaries and freedom to roam. Unconfined dogs had unrestricted movement, potentially posing safety and regulation challenges. We also asked respondents about access to veterinary care if a veterinary establishment existed in their location (with options: Yes, No, and I am not aware), considering that rabies vaccines in Nigeria are commonly administered in veterinary hospitals [17]. Dog vaccination status, indicated by a binary response, was further verified by asking owners to produce a valid vaccination certificate. Finally, we used a collection of questions to target each responder's knowledge about rabies. These questions asked whether responders were familiar with the word ‘rabies’ (binary), the mode of rabies virus transmission (binary), the types of hosts susceptible to rabies (ordinal), and medical options in the event of a dog bite (binary). For individuals who stated that their dogs were not vaccinated against rabies, follow-up categorical questions were asked about why they were not vaccinated. The types of categories that respondents could choose included vaccine cost, not knowing how to access the vaccine, or that the vaccine was unimportant. We also asked about individual dog information, such as breed (categorical), age (discrete), sex (nominal), and the primary use of the dog (categorical). The survey also included information on the number of dogs in each household (discrete) and the method of feeding (categorical). For analysis purposes, we reclassified the number of dogs in a household into two groups (1 and > 1). A total of ten houses were surveyed in each street chosen.

We used additional information to account for certain sociocultural factors influencing the human-animal relationship, specifically those that drove the willingness to vaccinate a dog against rabies [16,23]. Given that a recent study demonstrated variation in language about dog ownership and rabies knowledge [48], we classified responses based on the geographical region of respondents. Based on our knowledge of Nigeria, there are more Hausa speakers in the Northwest (Kaduna) and Northcentral (FCT) and more Igbo speakers in the Southeast (Enugu and Anambra). Due to the sensitivity of religion in Nigeria and the fact that certain religions consider dogs to be impure, we excluded religion from our analysis.

A recent study in Nigeria also demonstrated a correlation between urbanisation and access to health services [31]. To account for the effect of the location of dog owners on dog vaccination, we obtained the urban extent grid raster map (geospatial dataset of urban/rural extents) from the Global Rural-Urban Mapping Project [49]. We overlayed the coordinates of the households collected during the survey on the map and extracted values using the R programming language (version 4.2.1). We converted the household coordinates to a spatial object using the sf package [50]. The raster values corresponding to the household locations were extracted using the raster:extract function, where 1 = rural and 2 = urban.

### 2.4 Instruments

We administered the survey orally in English, Hausa, Yoruba and Igbo, and the interviewers recorded the responses on electronic handheld devices using the KoboToolbox [51]. The survey was developed in English in collaboration with experts in the field (Dr Philip P. Mshelbwala, Dr. Nicholas Clark, Prof. Charles E. Rupprecht, Prof. J. Scott Weese, Dr. Osinubi Modupe and Dr. Emanual Njoga).

### 2.5 Causal framework

**Fig. 1:**
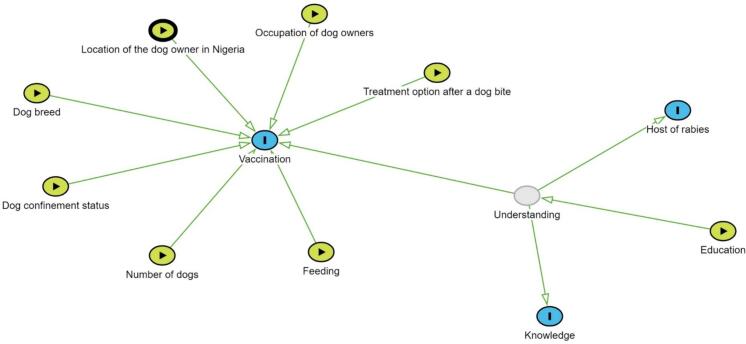
A Directed Acyclic Graph (DAG) illustrates the hypothetical variations in rabies vaccination status. The observed variables consisted of dog breed, primary use of the dog, number of dogs in the house, the dog's confinement status, how the dog is fed (feeding), occupation of the dog owner and treatment option following a dog bite, location of the dog owner in Nigeria and a latent variable representing the respondents' general comprehension of rabies (Understanding). Education directly influences latent understanding. The blue circles represent outcome variables, the green circles represent exposures (i.e., predictor variables), and the grey circles represent unobserved variables. Generally, green lines represent the front door path or the causal effect of interest. (For interpretation of the references to colour in this figure legend, the reader is referred to the web version of this article.)

We designed two causal diagrams in this study to inform data collection and analysis using DAGitty v3. The first described our proposed causal framework for relationships among variables and their potential influences on rabies vaccination decisions. Our DAG illustrated how rabies vaccination status was hypothesised to vary with an observed confounder education, observed variables including dog breed, the primary use of dog, confinement status of the dog, how the dog is fed, occupation of the dog owner, number of dogs owned by a household, location of dog owner (urban or rural), option for medical intervention/prophylaxis in the event of a dog bite (traditional vs contemporary), region where a dog owner is located in Nigeria and a latent variable describing respondents' overall understanding of rabies. While this latent understanding cannot be directly observed, in our study, we proposed that it manifested in each respondent's response to a set of familiarity about rabies, including familiarity with the word ‘rabies’, the modes of transmission of rabies virus, and the types of hosts that can be affected by rabies. The diagnostic checks suggested suboptimal convergence indicated by an elevated R-hat value beyond acceptable thresholds, coupled with erratic and non-stationary behaviour in the trace plot for the education variable on vaccination. These findings suggested potential challenges in achieving convergence, highlighting the necessity for further investigation and refinement in the modelling process. We then constructed new DAGs that made similar assumptions with education having a direct effect only on latent understanding and without rural and urban locations (Fig. 1).

**Fig. 2:**
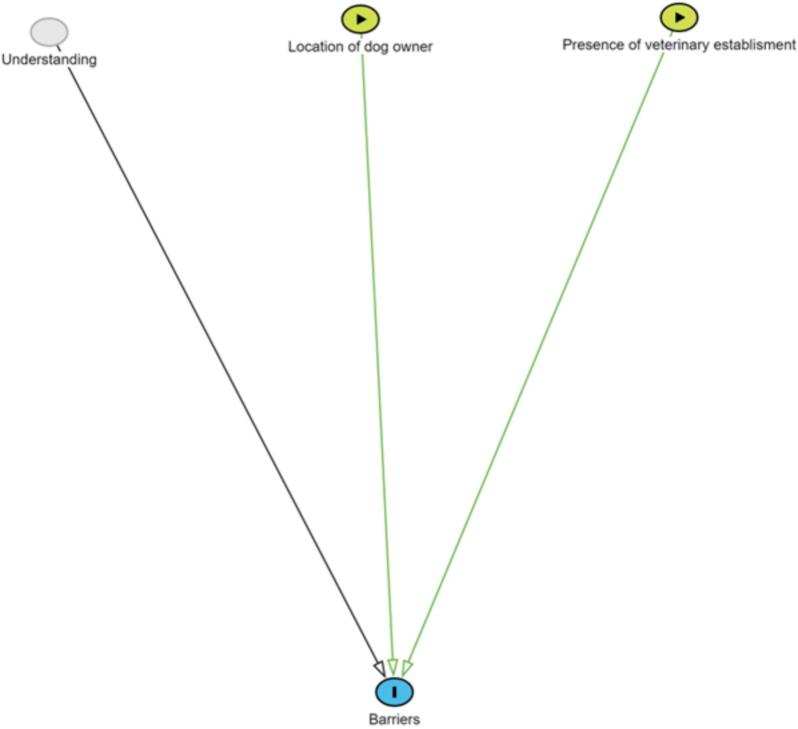
Directed Acyclic Graph (DAG) visually represents our proposal that the primary barrier preventing dog owners from getting their dogs vaccinated against rabies is determined by three factors: their latent understanding of rabies, accessibility of veterinary services in their local area, and the geopolitical zone in which a dog owner is located (location of dog owner). Green lines represent the front door path or the causal effect of interest, while black lines represent neither. (For interpretation of the references to colour in this figure legend, the reader is referred to the web version of this article.)

The second DAG illustrated our assumptions about the relationships between respondent variables and their stated barriers to vaccination. This was the focus of a categorical question that asked respondents to state why they did not vaccinate their dogs. Consequently, this DAG only applied to the subset of respondents whose dogs were not vaccinated. We posited that a person's action depended on their latent understanding of rabies, education level, the dog owner's geopolitical zone as a proxy for cultural influences, and the owner's location (urban or rural area). Our rationale for these dependencies was that respondents who were more aware of rabies were likely to select cost or access as the primary barrier to vaccination, whereas those who were less aware might choose a response that indicated mistrust in rabies vaccines. However, diagnostic assessments for this model revealed suboptimal convergence, with an elevated R-hat value exceeding acceptable thresholds and erratic, non-stationary behaviour observed in the trace plot for the effect of education and urban/ rural location on barriers to dog vaccination. These findings suggested potential challenges in achieving convergence, highlighting the necessity for further investigation and refinement in the modelling process. Consequently, we developed another DAG assuming that access to vaccination is determined by the dog owners' categorical responses regarding the presence of a veterinary establishment in their locality. Given that dog rabies vaccine is commonly delivered through veterinary clinics in Nigeria [12,17] —the response options being “Yes, there is a veterinary establishment,” “No, there is no veterinary establishment,” and “I do not know”, with their latent understanding of rabies and the geopolitical zone where the dog owner was located (location of the dog owner) (Fig. 2).

### 2.6 Analysis

We built a joint probabilistic model using Stan version 2.26.1 in R Studio environment [52] to target our primary estimand (How does latent knowledge of rabies influence a dog owner's decision to vaccinate against rabies?) and to make inferences about the types of barriers that dog owners see as influencing their decisions to vaccinate.

In this study, our unit of analysis was the individual household that owned a dog. The first component of our model used the observed vector of binary responses to familiarity with rabies (*Familiarity*) as outcome variables assumed to be drawn from Bernoulli distributions with unknown parameters (*p*_*i*_). We modelled the *p*_*i*_'s as directly depending on the latent understanding variable (Understanding) and intercept terms (α_*i*_) to capture systematic variation in average responses to each familiarity question:

*For i in 1,2 … I familiarity with rabies questions*

*Familiarity*_*i*_ ∼ Bernoulli(*p*_familiarity[i]_)

logit(*p*_familiarity[i]_) = α_familiarity[i]_ + *understanding*

α_familiarity_ ∼ Normal (−0.25, 1)

The second component was also designed to improve inferences about the latent understanding variable. Our outcome of interest was an ordered response to a survey question about what kinds of host species are susceptible to rabies virus (host). Respondents who answered correctly (by answering the question that rabies affects both humans and animals) scored 3, those with one correct host scored 2, and those who answered ‘neither humans nor animals’ scored 1. We used an ordinal regression model that depended on latent knowledge (understanding) as a predictor. Ordinal outcomes can be modelled by introducing a latent continuous variable that is related to the observed outcomes via a Categorical observation model with a cumulative logit link function, where a set of internal cutpoints (*c*_*k*_) partitions the log-cumulative-odds of the *K* ordered response categories. We used a principled Dirichlet prior model on internal cutpoints, ensuring a robust prior model when ordinal data are weakly informative in some or all possible response categories [53].

*For k in 1,2 … K ordered rabies host response categories*

*host* ∼ Categorical(*p*_host_)

*p*_host [1]_ = *q*_1_

*p*_host[*k*]_ = *q*_*k*_ – *q*_*k*-1_ for *K* > *k* > 1

logit(*q*_*k*_) = *c*_*k*_ – *understanding*

*c*_*k*_ ∼ Dirichlet(*K*)

The latent *Understanding* variable was assumed to follow a normal distribution in which the mean was modelled using a linear predictor to capture our proposed dependency on education. The variance parameter (*σ*_*understanding*_) was not identifiable at values approaching 0 (which would result in the latent understanding variable perfectly matching the measured education variable), so we specified an informative uniform prior density to capture our belief that a person's knowledge of rabies was strongly, but not solely, correlated with education:

*understanding* ∼ Normal (μ_understanding_, *σ*_*understanding*_)

*μ*_*understanding*_ = β_*understanding*_ · education

*σ*_*understanding*_ ∼ Uniform (0.5, 1)

β_*understanding*_ ∼ Normal (0.25, 1)

The final component was also built with the assumption that the observed vector of outcome dog vaccination decisions (vaccinate) was drawn from a Bernoulli distribution with unknown parameters(p) and depended on linear, additive effects of latent knowledge (understanding), education levels (education), breed of dogs (breed), dog use, number of dogs, how a dog is fed (feeding), dog confinement statuses and the location of the dog owner. In cases where households had multiple dogs, we classified them as vaccinated when an equivalence of 70% of their dogs had received rabies vaccinations, supported by valid vaccination certificates as evidence.

vaccinate ∼ Bernoulli(*p*_vaccinate_)

logit(*p*_vaccinate_) = α_vaccinate_ + β_vaccinate_ · X_vaccinate_

X_vaccinate_ = [*understanding*^T^ *Dog use* ^T^ *confinement status of the do*g ^T^ *Number of dogs* ^T^ Dog *breed* ^T^ *Feeding* ^T^ *Occupation of dog owner* ^T^ *Location of dog owner* ^T^ *Treatment following a dog bite* ^T^]

α_vaccinate_ ∼ Normal (−0.75, 1)

β_vaccinate_ ∼ Normal (0.25, 1)

For the subset of the respondents with unvaccinated dogs, we built a multinomial regression model for barriers to dog vaccination. For the first model, barriers to dog vaccination depended on latent understanding, access to veterinary services and the location of the dog owner. Our outcome of interest was a choice of three unordered categorical options specifying the primary reasons given by the dog owners for failure to vaccinate their dogs against rabies (Barriers), including cost, not knowing how to get rabies vaccine, and not thinking the vaccine was essential and the location of the dog owner in Nigeria (Kaduna, FCT, Anambra and Enugu). Given language variation across northern and southern Nigeria, information was gathered using different local options.

barrier ∼ Categorical(*p*_barrier_)

logit(*p*_barrier_) = β_barrier_ · X_barrier_

X_barrier_ = [*understanding*^T^ *Existence of veterinary establishment*^T^ *Location of the dog owner*^T^].

β_barrier_ ∼ Normal (0,1)

To ensure our prior choices excluded unrealistic behaviours, we used previous reports on rabies that explored knowledge, attitudes, practices, and dog vaccination to simulate the expected data-generating process (Supplementary file 2).

## 3 Results

### 3.1 Demographic characteristics of survey participants and dog ownership practices around rabies vaccination

A total of 4162 dog owners were surveyed between June and December 2022, of which 53% (2193/4162) reported dogs that were vaccinated against rabies within the last year. Table 1 summarises the results obtained during the survey.

**Table 1:** Demographic characteristics of survey participants, including individual dog information and ownership practices.

Characteristic	*N* = 4162	Vaccinated (%)	Unvaccinated (%)
Have you heard about rabies?			
Yes	2905	1751 (60.3)	1154 (39.7)
No	1257	442 (35.1)	815 (64.9)

Bite from rabies-infected dogs can transmit rabies, True or False			
False	2807	1474 (52.5)	1333 (47.5)
True	1355	636 (46.9)	719 (53.1)

Rabies affects which of the following?			
I do not know	806	276 (34.2)	530 (65.8)
Only humans or only animals	1342	692 (51.6)	650 (48.4)
Both humans and animals	2014	1225 (60.8)	789 (39.2)

What is your education level?			
None	554	177 (31.9)	377 (68.1)
Primary/Secondary	1980	984 (49.7)	996 (50.3)
Tertiary	1628	1032 (63.4)	596 (36.6)

What is the primary use of the dog?			
Security	2305	1293 (56.1)	1012 (43.9)
Pet	947	530 (55.9)	417 (44.1)
Breeding and hunting	910	370 (40.7)	540 (59.3)

How many dogs are in your household?			
One	2422	1373 (56.7)	1049 (43.3)
More than one	1740	820 (47.1)	920 (52.9)

Is there a veterinary establishment in your location?			
Yes	2242	1482 (66.1)	760 (33.9)
No	1349	562 (41.7)	787 (58.2)
I do not know	571	149 (26.1)	422 (73.9)

Is your dog under confinement?			
Yes	1677	1217 (72.6)	460 (27.4)
No	1620	590 (36.4)	1030 (63.6)
Partial confinement	820	378 (46.1)	442 (53.9)

Dog breed			
Native	2703	1220 (45.1)	1483 (54.9)
Crossbreed/exotic	1459	973 (66.7)	486 (33.3)

What is your occupation?			
Student	1275	726 (56.9)	549 (43.1)
Civil servant	779	542 (69.6)	237 (30.4)
Private sector	2108	925 (43.9)	1183 (52.1)

What treatment option would you opt for in the event of a dog bite?			
Contemporary	3050	1783 (58.5)	1267 (41.5)
Traditional	1112	410 (36.9)	702 (60.1)

Location of the dog owner			
FCT	1661	1071 (64.5)	590 (35.5)
Kaduna	1247	690 (55.3)	557 (44.7)
Anambra and Enugu	1254	432 (34.4)	822 (55.6)

Private sector (unemployed and individuals running their own businesses).

### 3.2 Posterior distribution of estimated median effects of familiarity and host of rabies questions along the latent understanding of rabies

Fig. 4 illustrates the estimated posterior distribution of the median effects of factors associated with vaccination. In terms of latent understanding and observed response that contributed to it, there was a positive correlation between respondents who knew that rabies affects both humans and animals and latent understanding. There was a negative correlation between latent understanding and respondents who were only aware of one host of rabies virus transmission. There was a positive correlation between respondents who had heard about rabies and were aware of the transmission pathway and latent understanding and a negative correlation between those who had not heard about or were aware of the transmission pathway.

### 3.3 Posterior distribution of estimated median effects of factors associated with vaccination, including latent understanding of rabies

**Fig. 3:**
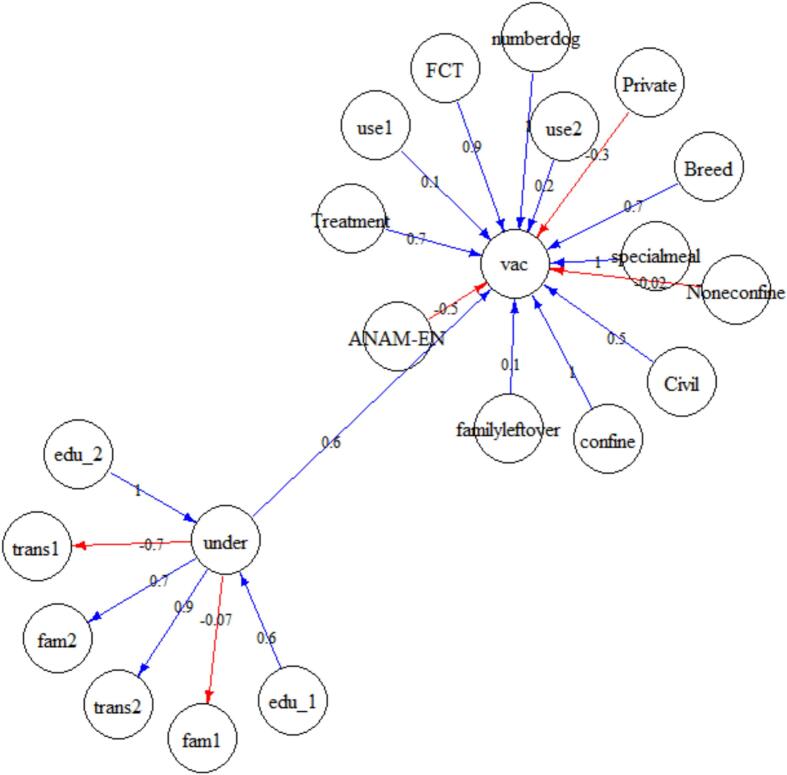
Posterior median estimates of factors associated with the vaccination and non-vaccination of dogs against rabies, including latent understanding of rabies (referred to as “under”). This understanding was a product of two distinct awareness states: Trans2 (indicating respondents who knew that rabies affected both humans and animals) and Trans1 (representing respondents who were only aware of one host of rabies virus transmission), Fam1 (those who had heard about rabies and were aware that rabid dogs could transmit) and Fam2 (those who had not heard about rabies and were unaware of the transmission pathway). There was a positive correlation between latent understanding (under) and higher levels of education (edu 1 and edu2), compared to no education. Our results suggested that dog vaccination (vac) was associated with the owner's latent knowledge of rabies (under), civil servant employment (Civil), residence in the FCT, ownership of a single dog (numberdog), dog confinement (confine), providing special meals to the dog (special), dog breed (breed) and preference for contemporary medical intervention. Conversely, non-vaccination was associated with private employment (Private), residing in Anambra and Enugu states (ANAM_EN), owning multiple dogs, non-confinement (Noneconfine), and a preference for traditional treatment after a dog bite.

Posterior distributions of the median effect of factors associated with dog vaccination revealed that the probability of dog vaccination was positively associated with latent understanding, higher levels of education, civil service employment, residence in the FCT, ownership of a single dog, dog confinement, providing special meals to the dog, dog breed, and preference for contemporary treatment. Conversely, non-vaccination was associated with private employment, residing in Anambra and Enugu states (ANAM_EN), owning multiple dogs, non-confinement, and a preference for traditional treatment after a dog bite (Fig. 3).

### 3.4 Posterior mean, odd ratio, standard deviation, credible intervals, and convergence diagnostics for estimated effects on the logit (probability of vaccination) among dog owners

Posterior distributions revealed that the probability of dog vaccination was positively associated with the owner's latent knowledge of rabies [Odds Ratio (OR), 95% credible interval (Crl) 2.46 (2.05–3.01)], civil service employment compared to private employment [1.79, OR, Crl (1.44–2.26)], residence in the FCT [OR, 1.80, Crl (1.42–2.29)] compared to Anambra and Enugu, providing special meals to dog compared to allowing dogs to search for food [OR, 2.40 Crl(2.60–3.07)], and confined dogs [OR, 2.54, Crl (2.08–3.10)] compared to free-roaming.

Conversely, non-vaccination was positively associated with owning multiple dogs [OR, 0.86 Crl (0.73–1.02)] compared to a single dog and with a preference for traditional treatment in the event of a dog bite compared to contemporary medicine [OR, 0.44, Crl (0.46–0.52)] (Supplementary file 3).

### 3.5 Estimated posterior distribution of factors associated with canine rabies vaccination and barriers to dog vaccination among owners in Nigeria

**Fig. 4:**
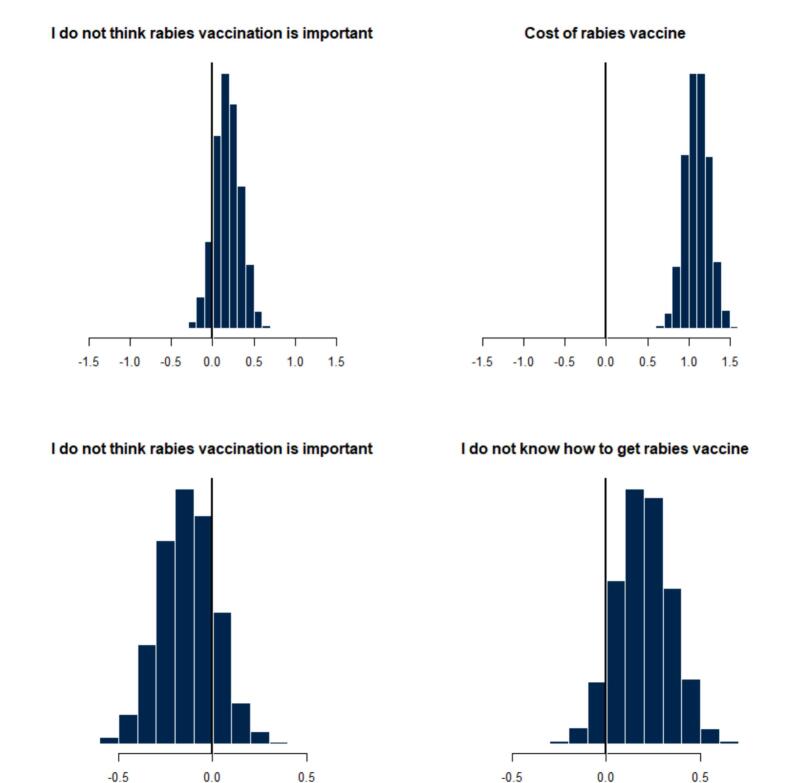
The posterior distribution of barrier choices among dog owners suggests those who reported the presence of a veterinary establishment in their location were more likely to identify cost as the primary barrier (Top right), while those who reported the absence of a veterinary establishment in their location were more likely to identify access (I do not know how to get rabies vaccine) (Bottom right).

**Fig. 5:**
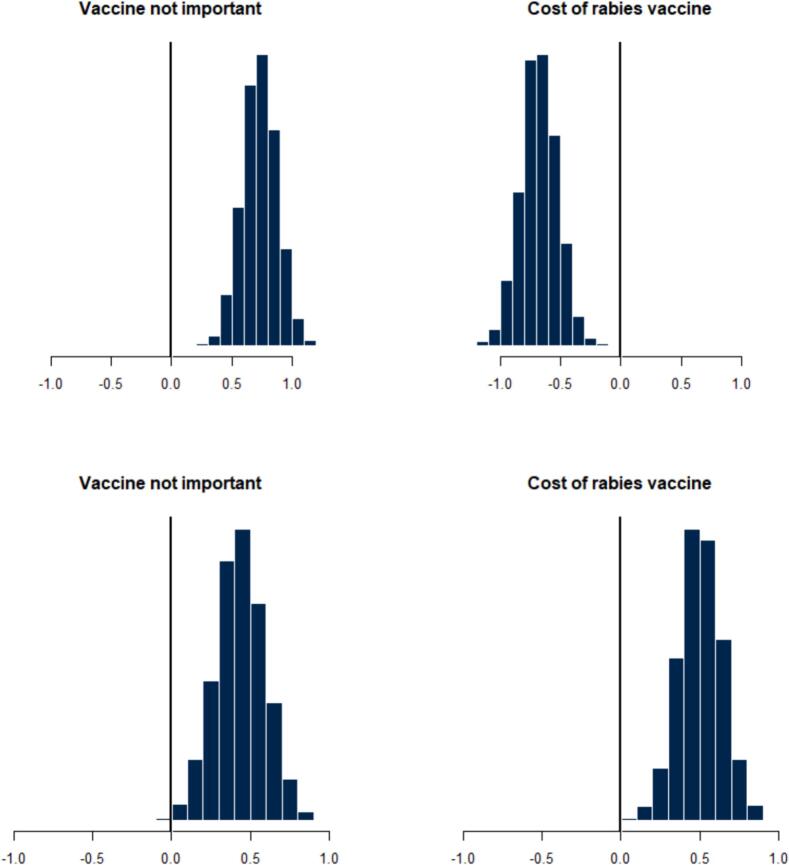
The posterior distribution of the locations of dog owners suggests that those in FCT had a higher probability of selecting ‘rabies vaccine not important’ as the primary barrier (Top left) and a lower probability of choosing ‘cost’ (Top right), while respondents in Anambra and Enugu had a higher probability of choosing ‘rabies vaccine not important’ (Bottom left) and ‘cost’ as major barriers (Bottom right).

The posterior distributions of barrier choices indicated that dog owners in areas with veterinary establishments cited cost as a barrier (Fig. 4), while access was the major barrier for those without a veterinary establishment (Fig. 4). Hesitancy (expressing the belief that the rabies vaccine is not important) posed a challenge for those in the FCT (Fig. 5), while cost was the primary barrier for dog owners in Anambra and Enugu states (Fig. 5). Latent understanding was negatively associated with two barrier choices, including the perception that the rabies vaccine was unimportant [Mean, −1.149, 75% - 95 Crl (−1.054, −0.886)] and concerns about the cost of the vaccine [Mean-0.88 Crl(−1.054, −0.886)].

## 4 Discussion

This study represented a significant advancement over previous investigations, as it goes beyond the commonly used stepwise selection process to address confounding factors [16,54]. Instead, we employed a causal diagram framework, specifically DAGs [27], to thoroughly analyze factors influencing dog vaccination in Nigeria. Using DAGs, we established strong evidence of the associations between various factors and factors associated with canine vaccination. The current study is the first multi-state investigation conducted in Nigeria. It provided evidence of regional variations in factors associated with vaccination and non-vaccination practices, which is crucial for implementing the recently developed national strategic plan for rabies prevention and control.

In this study, latent understanding was associated with dog vaccination. This finding aligned with a study conducted in the Philippines, which aimed to assess dog owners' willingness to pay for dog vaccination and revealed that knowledge about the signs of rabies was positively correlated with their willingness to pay [55]. This further underscored the challenges faced in rabies control efforts in Nigeria and emphasised the crucial importance of raising awareness. Although the annual observance of World Rabies Day on 28 September has contributed to increased awareness, additional and sustained routine efforts on a larger scale are required to increase engagement about rabies and the importance of responsible pet ownership, including the provision of vaccination.

Furthermore, addressing the language barriers issue was vital, as most of these events were conducted in English, leaving non-English speakers uninformed. Indeed, our model suggested a high probability of vaccination in the FCT compared to Anambra and Enugu. Community-based enlightenment programs catering to diverse linguistic and cultural backgrounds can bridge this gap and ensure broader participation and understanding.

Our study also observed an association between dog vaccination and being employed as a civil servant instead of individuals in private employment/unemployed. Given the context of Nigeria, where unemployment rates are relatively high [56], a notable income gap exists between these two groups. Civil servants earn comparatively higher incomes than privately employed/unemployed individuals. Indeed, our results suggested that cost remains a major obstacle to dog vaccination. Previous disease control initiatives in Nigeria, such as the Expanded Programme on Immunization (EPI), provided free vaccinations for children nationwide, utilizing dedicated days for regular immunization over an extended period, engaging the communities, stakeholders, and partners [57]. Similarly, for Nigeria to effectively work towards eliminating canine-mediated rabies by 2030, it is advisable to consider the implementation of widespread, complimentary mass dog vaccination campaigns. This approach is recommended over the current practice of relying on veterinary clinics and sporadic vaccination efforts [17].

Furthermore, education being a prerequisite for recruitment into the civil service in Nigeria, it is plausible that civil servants may have higher levels of education, potentially contributing to a better understanding of the importance of rabies vaccination [58]. Indeed, a study reported that civil servants in Taraba, Nigeria, were 4.8 times more likely to have good knowledge about rabies than other professional groups [59]. This heightened awareness might motivate civil servants to prioritise vaccinating their dogs against rabies. This suggested a positive association between latent understanding and education levels. These factors should be considered when developing and implementing widespread vaccination initiatives in Nigeria, particularly in identifying communities that require additional resources and educational support.

Respondents in FCT exhibited a higher probability of vaccinating their dogs against rabies than those in Anambra and Enugu states. This discrepancy might be due to the cosmopolitan nature of the FCT, drawing a population that includes diplomats and individuals from diverse, affluent, and educated backgrounds. Moreover, in contrast to the Northern region, veterinary units responsible for administering animal vaccines, including rabies vaccines, often face staffing shortages in the Southeastern region [60]. This finding underscores the importance of addressing these disparities in veterinary resources and public awareness efforts to combat rabies effectively.

Our model results suggested respondents in FCT were more likely to select ‘rabies vaccine as not important’ as the primary barrier. In a recent national survey of over 2000 dog owners in the USA, 52% demonstrated some level of canine vaccination hesitancy [61]. The perception that rabies vaccines were not available among owners in Nigeria emphasises the critical need for community engagement initiatives to build trust.

Moreover, our multinomial model suggests access to rabies vaccine was a major barrier among respondents without a veterinary clinic. This is consistent with the model of rabies vaccine delivery via veterinary clinics in Nigeria [62]. Indeed, the Nigerian Veterinary Medical Association (NVMA) recently attributed the lack of veterinary establishments in remote areas as one of the key drivers of rabies in the country [63]. As of 2018, only over 5000 veterinarians were servicing over 200 million Nigerians across 774 local government areas [64].This underscores the imperative to enhance access to rabies vaccination and address the scarcity of veterinary services.

The present study further identified that dog breed, confinement status, the number of dogs in a household, and how a dog was fed were associated with dog vaccination. These findings aligned with previous research conducted in various regions of Africa and could be attributed to several factors. [16,47,65]. The association between non-vaccination against rabies and owning more dogs highlights the practical challenges of having multiple dogs in the current Nigerian context, particularly considering security and poverty factors [46]. While dog owners may desire to own more dogs for security reasons [16,46], it is crucial to recognize the potential consequences of caring for these dogs. This scenario could contribute to the increased population of free-roaming dogs, presenting additional public health and safety concerns. Indeed, our results indicated that dog vaccination was associated with confinement and providing special meals to a dog. This finding aligned with the results of a recent hospital study in Nigeria, which demonstrated a higher probability of deaths among individuals bitten by free-roaming dogs compared to confined dogs [17]. It emphasised the necessity of prioritising interventions that promote responsible dog ownership, thus reducing the risk of fatal outcomes from dog bites.

As with any survey, it is crucial to acknowledge limitations of this study. While the structured sampling method was implemented to minimize bias, there remains a possibility of sampling bias. The determination of vaccination status relied on both querying dog owners and examining vaccination records. However, recall bias or misinformation may have introduced some level of influence on the results. Nevertheless, these concerns were deemed minimal overall. Other limitations included the exclusion of spiritual determinants that may influence the human-animal relationship, factors such as the maximum amount dog owners are willing to pay, and the barriers they surmounted before vaccinating their dogs.

The findings of this study shed light on several areas that warrant further research. Specifically, there is a need to investigate the maximum amount dog owners are willing to pay to vaccinate their dogs. This additional research could serve as a crucial guide for policymakers in designing more effective and sustainable strategies to improve vaccination rates and ensure broader access to rabies control measures. Ideally, although vaccination should be free, understanding the cost considerations of dog owners facilitate the implementation of targeted interventions that align with the economic realities of different communities, thus promoting higher vaccination coverage and contributing to the overall success of rabies eradication efforts in Nigeria. Moreover, considering the finite resources available for public health initiatives, spatial models could be employed to identify communities with the lowest vaccination rates. This valuable information can guide the implementation of targeted interventions, directing resources and efforts towards areas with the greatest need. By focusing on communities with lower vaccination rates, policymakers can maximize the impact of their interventions and significantly contribute to the overall success of rabies prevention and control efforts in Nigeria.

In conclusion, the present study offered valuable insights into the factors influencing dog vaccination in Nigeria. Our work provided a solid foundation for evidence-based policymaking to enhance rabies prevention and control efforts and strive towards the ambitious goal of eliminating human rabies deaths by 2030.

## CRediT authorship contribution statement

**Philip P. Mshelbwala:** Conceptualization, Data curation, Formal analysis, Investigation, Methodology, Software, Validation, Visualization, Writing – original draft, Writing – review & editing. **Charles E. Rupprecht:** Writing – review & editing. **Modupe O. Osinubi:** Methodology, Writing – original draft. **Emmanuel O. Njoga:** Investigation. **Terese G. Orum:** Investigation. **J. Scott Weese:** Writing – review & editing. **Nicholas J. Clark:** Conceptualization, Formal analysis, Methodology, Supervision, Writing – review & editing.

## Declaration of competing interest

The authors declare no conflicts of interest. The findings and conclusions in this report are those of the authors and do not necessarily represent the views of the Centers for Disease Control and Prevention.

## Data Availability

Data will be made available on request.

## Appendix A Supplementary data

Supplementary material 1 Image 1

Supplementary material 2 Image 2

Supplementary material 3 Image 3
