# Supplementary material for: Factors influencing canine rabies vaccination among dog-owning households in Nigeria
Source: One Health. 2024 May 10;18:100751. doi: 10.1016/j.onehlt.2024.100751 (PMC11141449; doi:10.1016/j.onehlt.2024.100751)
Supplement: Supplementary file 1 — Supplementary material 1 [file mmc1.docx]

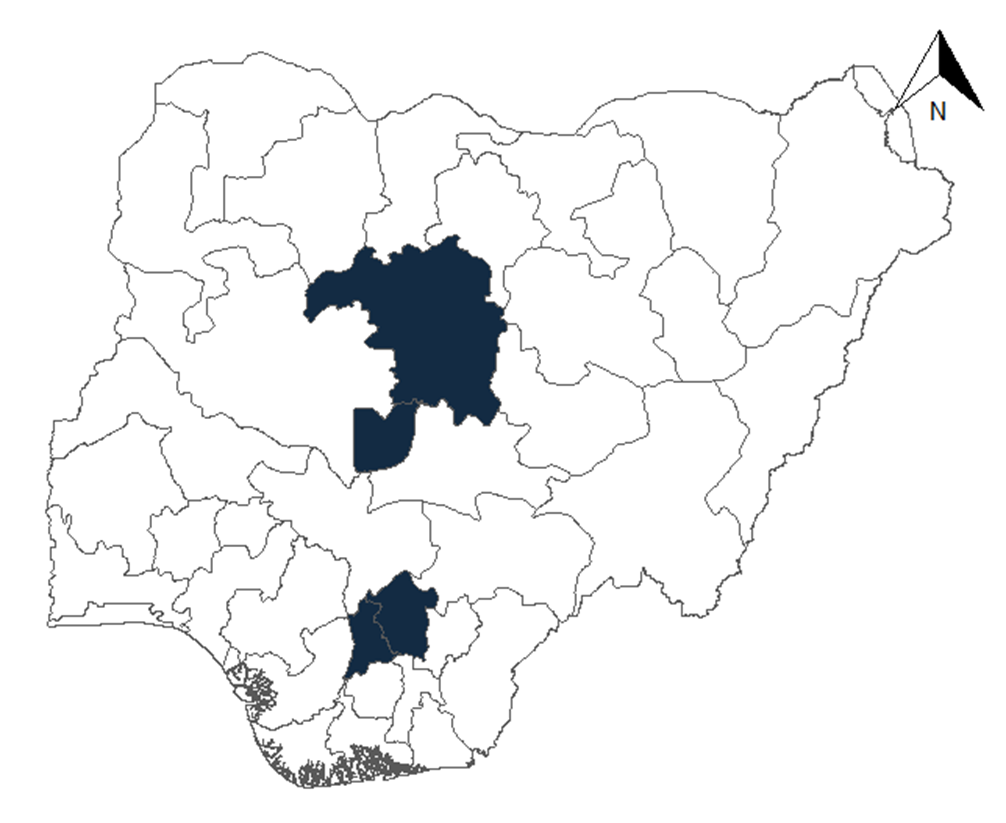


Supplementary file 1: The black areas on the map indicate the geographical locations within Nigeria where the study was conducted. These locations encompass Kaduna (Northwest), the FCT (North Central), and Anambra and Enugu (Southeast).
