# Supplementary material for: Factors influencing canine rabies vaccination among dog-owning households in Nigeria
Source: One Health. 2024 May 10;18:100751. doi: 10.1016/j.onehlt.2024.100751 (PMC11141449; doi:10.1016/j.onehlt.2024.100751)
Supplement: Supplementary file 2 — Supplementary material 2 [file mmc2.docx]

# Global rstan options

library(rstan)

rstan_options(auto_write = TRUE)

options(mc.cores = parallel::detectCores())

parallel:::setDefaultClusterOptions(setup_strategy = "sequential")

# Global colour scheme

c_light <- c("#DCBCBC")

c_light_highlight <- c("#C79999")

c_mid <- c("#B97C7C")

c_mid_highlight <- c("#A25050")

c_dark <- c("#8F2727")

c_dark_highlight <- c("#7C0000")

#### Step 1: Simulate data under expected data generating process ####

# We start by simulating education outcomes for a population of people

# Number of simulated people

N = 1000

# Simulated education, with more people expected to have lower education

set.seed(1111)

edu_true_factor <- as.factor(sample(c(1,2,3), prob = c(0.45, 0.35, 0.2), N, T))

edu_true <- as.matrix(as.data.frame(model.matrix(~ edu_true_factor - 1)))

head(edu_true)

colSums(edu_true)

# Simulated effects of education level on awareness

beta_aware_edu_true <- c(0, 0.35, 0.7)

# Simulated average awareness

alpha_aware_true <- -.25

# Simulated latent awareness is a function of education but also other sources of

# variation

aware_true <- alpha_aware_true + edu_true %*% beta_aware_edu_true + rnorm(N)

hist(boot::inv.logit(aware_true))

# Simulated correlated knowledges about rabies, with different average knowledge per

# question (people will likely know less about transmission than about the name rabies)

name_alpha <- 0.25

trans_alpha <- -0.65

name_knowledge_true <- rbinom(N, 1, boot::inv.logit(name_alpha + aware_true + rnorm(N)))

trans_knowledge_true <- rbinom(N, 1, boot::inv.logit(trans_alpha + aware_true + rnorm(N)))

sum(name_knowledge_true) / N

sum(trans_knowledge_true) / N

answers_true <- cbind(name_knowledge_true, trans_knowledge_true)

# Simulated vaccination outcomes

alpha_true <- -0.65

beta_edu_true <- c(0, 0.25, 0.5)

beta_aware_true <- 0.65

vaccinate_true <- rbinom(N, 1,

boot::inv.logit(alpha_true + edu_true %*% beta_edu_true +

aware_true * beta_aware_true))

# Simulated prevalence of vaccination

sum(vaccinate_true) / N

# Some visualisations and checks that incorrectly specified models will lead to poor inference

# First a function to visualise posterior parameter estimates

plot_glm_estimates = function(model, variable, truth){

# Simulate posterior estimates from a model fitted with maximum likelihood, which assumes

# the posterior is multivariate Gaussian

betas <- MASS::mvrnorm(1000, mu = coef(model), Sigma = vcov(model))

colnames(betas) <- names(coef(model))

# Plot and compare against the truth for the supplied parameter

hist(betas[,variable], xlab = paste0('Posterior estimates of ', variable, ' coefficient'),

main = '',

freq = F,

breaks = 30,

col = c_mid, border = c_mid_highlight)

abline(v = truth, lwd = 3, col = 'white')

abline(v = truth, lwd = 2, col = 'black')

}

# A linear model for the effect of education on the latent awareness variable correctly infers the

# causal structure

confint(lm(aware_true ~ edu_true_factor))

alpha_aware_true; beta_aware_edu_true

# A logistic regression that has access to the latent awareness variable will upwardly bias the

# estimated intercept and the estimated effect of awareness due to the missing positive effect of

# education on both awareness and on vaccination

aware_only <- glm(vaccinate_true ~ aware_true, family = 'binomial')

confint(aware_only)

alpha_true; beta_aware_true

plot_glm_estimates(model = aware_only,

variable = 'aware_true',

truth = beta_aware_true)

# But a model that now includes education results in better estimates of the intercept and the effect

# of awareness

full <- glm(vaccinate_true ~ aware_true + edu_true_factor, family = 'binomial')

confint(full)

alpha_true; beta_aware_true

plot_glm_estimates(model = full,

variable = 'aware_true',

truth = beta_aware_true)

# Of course we won't have access to the 'awareness' variable when modelling our real data so

# we will need more complex joint models to estimate it and its effect on vaccination

# Gather data for modelling

model_data <- list(N = N,

vaccinate = vaccinate_true,

knowledge_answers = answers_true,

K = NCOL(answers_true),

name_knowledge = name_knowledge_true,

trans_knowledge = trans_knowledge_true,

edu = edu_true)

#### Step 2: explore model configurations for capturing the model's latent structure ####

# In the first model, we will assume very little prior knowledge and use traditional vague priors on

# any coefficients. We will also not include a model for vaccination yet so we can explore how

# our model assumptions translate to expected vaccination rates

mod1 <- "data {

int<lower=0> N; // number of data points as an integer

int<lower=0,upper=1> vaccinate[N]; // binary outcome vector

matrix[N, 3] edu; // education level is a matrix of categorical outcomes

int<lower=0> K; // number of knowledge questions

int<lower=0, upper=1> knowledge_answers[N,K]; // binary answers to knowledge questions

}

parameters {

vector[N] aware; // latent awareness vector

real alpha; // intercept term for vaccination

real beta_aware; // effect of awareness on vaccination

vector[K] alpha_questions; // intercept terms for knowledge responses

vector[2] beta_edu_raw; // raw effect of education on vaccination

}

transformed parameters {

// Need first level of beta_edu coefs to be zero

vector[3] beta_edu;

for (i in 2:3){

beta_edu[i] = beta_edu_raw[i-1];

}

beta_edu[1] = 0;

}

model {

alpha ~ normal(0, 10);

beta_aware ~ normal(0, 10);

beta_edu_raw ~ normal(0, 10);

// Latent awareness variable with no presumed relationship to education

aware ~ normal(0, 1);

// Model for question responses

alpha_questions ~ normal(0, 10);

for (k in 1:K){

knowledge_answers[,k] ~ bernoulli_logit(to_vector(alpha_questions[k] + aware));

}

// Model for vaccination is excluded

// vaccinate ~ bernoulli_logit(alpha + beta_aware * aware + edu * beta_edu);

}

generated quantities {

// Posterior predictions of vaccination

int<lower=0,upper=1> vaccinate_preds[N];

vector[N] prob_vacc;

prob_vacc = inv_logit(alpha + beta_aware * aware + edu * beta_edu);

vaccinate_preds = bernoulli_rng(prob_vacc);

// Posterior predictions for the knowledge questions

int<lower=0,upper=1> name_preds[N];

vector[N] prob_name;

prob_name = inv_logit(to_vector(alpha_questions[1] + aware));

name_preds = bernoulli_rng(prob_name);

int<lower=0,upper=1> trans_preds[N];

vector[N] prob_trans;

prob_trans = inv_logit(to_vector(alpha_questions[2] + aware));

trans_preds = bernoulli_rng(prob_trans);

}

"

# Condition the model on the observed data

fit1 <- stan(model_code = mod1,

data = model_data,

iter = 2000, chains = 4)

# Check convergence and other diagnostics (ignore Rhat warnings for parameters that are NaN)

source(paste0('https://raw.githubusercontent.com/',

'betanalpha/knitr_case_studies/',

'master/gaussian_processes/stan_utility.R'))

check_all_diagnostics(fit1)

# Check correlations among the simulated awareness vector and the truth; our goal is to

# get this as high as we can, suggesting our model is estimating latent awareness well

sim_awares <- extract(fit1, pars = 'aware')[[1]]

cors <- vector()

for(i in 1:dim(sim_awares)[1]){

cors[i] <- as.numeric(cor(sim_awares[i,], aware_true))

}

quantile(cors, probs = c(0.1, 0.5, 0.9))

# Define some prior pushforward summaries that we can use to interrogate the

# assumptions that our prior model is inherently implying

# Function for a prior predictive check for expected prevalences

ppc_prev = function(samples, expert_boundaries, variable = 'outcome'){

# Plot a histogram of the model's expected prevalence

expected_prevs = vector(length = dim(samples)[1])

for(i in 1:dim(samples)[1]){

expected_prevs[i] = sum(samples[i,]) / NCOL(samples)

}

hist(expected_prevs,

main = '',

breaks = 30,

xlim = c(0, 1),

xlab = paste0('Predicted prevalence of ', variable),

border = c_mid_highlight,

col = c_mid,

freq = F)

# Add expert-informed logical boundaries as lines

if(!missing(expert_boundaries)){

abline(v = expert_boundaries[1], lwd = 3,

col = 'white')

abline(v = expert_boundaries[1], lwd = 2.5,

col = 'black')

abline(v = expert_boundaries[2], lwd = 3,

col = 'white')

abline(v = expert_boundaries[2], lwd = 2.5,

col = 'black')

# Quantify how much of the probability mass is within the expert-informed

# boundaries

cat('Proportion in boundaries =',

round(length(which(expected_prevs >= expert_boundaries[1] &

expected_prevs <= expert_boundaries[2])) / length(expected_prevs), 2),

'\n')

}

}

# Function for a prior predictive check of expected probabilities

ppc_prob = function(samples, expert_boundaries, inv_logit = FALSE,

variable = 'outcome'){

# Plot a histogram of the model's expected probability

if(inv_logit){

# Convert to probability scale if required

expected_probs = matrix(NA, nrow = dim(samples)[1], ncol = dim(samples)[2])

for(i in 1:dim(samples)[1]){

expected_probs[i,] = boot::inv.logit(samples[i,])

}

} else {

expected_probs <- samples

}

hist(expected_probs,

main = '',

breaks = 30,

xlim = c(0, 1),

xlab = paste0('Predicted probability of ', variable),

border = c_mid_highlight,

col = c_mid,

freq = F)

}

# Perform a prevalence PPC for the vaccination status, using boundaries of 0 and 0.6

# (i.e. we expect true proportion of dogs in the study area that have been vaccinated to be somewhat low).

# Note that these boundaries should reflect the range that we would consider realistic, not any measurements

# that we have currently taken. So it is ok to keep them a bit wider than you might expect, as long as they

# exclude values that are completely at odds with what we would expect if we surveyed many different areas

# in the study region

pred_vacc <- extract(fit1, pars = 'vaccinate_preds')[[1]]

ppc_prev(pred_vacc, expert_boundaries = c(0, 0.6), variable = 'Rabies vaccination')

prob_vacc <- extract(fit1, pars = 'prob_vacc')[[1]]

# We would also expect the probability of vaccination to be somewhat skewed toward zero

ppc_prob(prob_vacc, variable = "vaccination against Rabies")

# Yikes!! This figure represents what is commonly known as the 'Devil's Horns', with probability

# masses heavily concentrating on the bounds. This is completely unrealistic but is how all

# 'vague' or 'uninformative' priors implicitly manifest.

# clearly our prior model is not at all consistent with our expectations.

# But before we go about altering priors, we whould have

# a look at the expected distribution for the latent 'awareness' variable. As this variable is a 'real'

# vector (continuous values), we convert it to probabilities using inverse logit

pred_aware <- extract(fit1, pars = 'aware')[[1]]

ppc_prob(pred_aware, variable = "'awareness' about Rabies", inv_logit = T)

# This variable is a bit hard to reason about, but in general we would expect

# overall awareness of rabies to be broadly distributed and perhaps a bit skewed toward zero.

# Model configuration 2 will use more realistic priors on coefficients, but still will not

# relate awareness to education

mod2 <- "data {

int<lower=0> N; // number of data points as an integer

int<lower=0,upper=1> vaccinate[N]; // binary outcomes

matrix[N, 3] edu; // education level is a matrix

int<lower=0> K; // number of knowledge questions

int<lower=0, upper=1> knowledge_answers[N,K]; // binary answers to knowledge questions

}

parameters {

vector[N] aware;

real alpha;

real beta_aware;

// In this model we now estimate latent awareness heterogeneity using a uniform

// distribution so that this variable's coefficients will be directly comparable to

// coefficients for effects of education and any other standardised predictors of

// vaccination

real<lower=0.5,upper=1> aware_sd;

vector[K] alpha_questions;

vector[2] beta_edu_raw;

}

transformed parameters {

// Need first level of beta_edu coefs to be zero

vector[3] beta_edu;

for (i in 2:3){

beta_edu[i] = beta_edu_raw[i-1];

}

beta_edu[1] = 0;

}

model {

// More informative priors for vaccination rates and effects of education

alpha ~ normal(-0.75, 1);

beta_aware ~ normal(0.25, 1);

beta_edu_raw ~ normal(0.25, 1);

aware_sd ~ uniform(0.5, 1);

// Latent awareness variable still does not consider education

aware ~ normal(0, aware_sd);

// Model for question responses is also more informative about averages

alpha_questions ~ normal(-0.25, 1);

for (k in 1:K){

knowledge_answers[,k] ~ bernoulli_logit(to_vector(alpha_questions[k] + aware));

}

// Model for vaccination is excluded

// vaccinate ~ bernoulli_logit(alpha + beta_aware * aware + edu * beta_edu);

}

generated quantities {

// Posterior predictions of vaccination

int<lower=0,upper=1> vaccinate_preds[N];

vector[N] prob_vacc;

prob_vacc = inv_logit(alpha + beta_aware * aware + edu * beta_edu);

vaccinate_preds = bernoulli_rng(prob_vacc);

// Posterior predictions for the knowledge questions

int<lower=0,upper=1> name_preds[N];

vector[N] prob_name;

prob_name = inv_logit(to_vector(alpha_questions[1] + aware));

name_preds = bernoulli_rng(prob_name);

int<lower=0,upper=1> trans_preds[N];

vector[N] prob_trans;

prob_trans = inv_logit(to_vector(alpha_questions[2] + aware));

trans_preds = bernoulli_rng(prob_trans);

}

"

# Condition the more domain-informative model on the simulated data

fit2 <- stan(model_code = mod2,

data = model_data,

iter = 2000, chains = 4)

check_all_diagnostics(fit2)

# Check correlations among the simulated awareness and the truth

sim_awares <- extract(fit2, pars = 'aware')[[1]]

cors <- vector()

for(i in 1:dim(sim_awares)[1]){

cors[i] <- as.numeric(cor(sim_awares[i,], aware_true))

}

quantile(cors, probs = c(0.1, 0.5, 0.9))

# Repeat posterior checks

pred_vacc <- extract(fit2, pars = 'vaccinate_preds')[[1]]

ppc_prev(pred_vacc, expert_boundaries = c(0, 0.6), variable = 'Rabies vaccination')

prob_vacc <- extract(fit2, pars = 'prob_vacc')[[1]]

ppc_prob(prob_vacc, variable = "vaccination against Rabies")

pred_aware <- extract(fit2, pars = 'aware')[[1]]

ppc_prob(pred_aware, variable = "'awareness' about Rabies", inv_logit = T)

# The prior model has improved but the awareness variable is still not what we'd expect;

# but what happens when we allow the latent awareness variable to be dependent on education and

# we use a more domain-informative prior for the mean awareness?

mod3 <- "data {

int<lower=0> N; // number of data points as an integer

int<lower=0,upper=1> vaccinate[N]; // binary outcomes

matrix[N, 3] edu; // education level is a matrix

int<lower=0> K; // number of knowledge questions

int<lower=0, upper=1> knowledge_answers[N,K]; // binary answers to knowledge questions

}

parameters {

vector[N] aware;

real alpha;

real alpha_aware;

real<lower=0.5,upper=0.75> aware_sd;

real beta_aware;

vector[K] alpha_questions;

vector[2] beta_edu_raw;

vector[2] beta_aware_edu_raw;

}

transformed parameters {

// Need first level of beta_edu coefs to be zero

vector[3] beta_edu;

vector[3] beta_aware_edu;

for (i in 2:3){

beta_edu[i] = beta_edu_raw[i-1];

beta_aware_edu[i] = beta_aware_edu_raw[i-1];

}

beta_edu[1] = 0;

beta_aware_edu[1] = 0;

}

model {

alpha ~ normal(-0.75, 1);

alpha_aware ~ normal(-0.5, 1);

beta_aware ~ normal(0.25, 1);

beta_edu_raw ~ normal(0.25, 1);

beta_aware_edu_raw ~ normal(0.25, 1);

aware_sd ~ uniform(0.5, 1);

// Latent awareness variable now depends on education; note this still needs to

// be zero-centred as its intercept will not be identifiable

aware ~ normal(edu * beta_aware_edu, 1);

// Model for question responses

alpha_questions ~ normal(-0.25, aware_sd);

for (k in 1:K){

knowledge_answers[,k] ~ bernoulli_logit(to_vector(alpha_questions[k] + aware));

}

// Model for vaccination still excluded

// vaccinate ~ bernoulli_logit(alpha + beta_aware * aware + edu * beta_edu);

}

generated quantities {

// Posterior predictions of vaccination

int<lower=0,upper=1> vaccinate_preds[N];

vector[N] prob_vacc;

prob_vacc = inv_logit(alpha + beta_aware * aware + edu * beta_edu);

vaccinate_preds = bernoulli_rng(prob_vacc);

// Posterior predictions for the knowledge questions

int<lower=0,upper=1> name_preds[N];

vector[N] prob_name;

prob_name = inv_logit(to_vector(alpha_questions[1] + aware));

name_preds = bernoulli_rng(prob_name);

int<lower=0,upper=1> trans_preds[N];

vector[N] prob_trans;

prob_trans = inv_logit(to_vector(alpha_questions[2] + aware));

trans_preds = bernoulli_rng(prob_trans);

}

"

# Condition the model that now includes the effect of education on awareness

fit3 <- stan(model_code = mod3,

data = model_data,

iter = 2000, chains = 4)

check_all_diagnostics(fit3)

# Check estimated parameters against truths

stan_hist(fit3, 'beta_aware_edu')

summary(fit3, 'beta_aware_edu')$summary[,1:3]

beta_aware_edu_true

# Check correlations among the simulated awareness and the truth

sim_awares <- extract(fit3, pars = 'aware')[[1]]

cors <- vector()

for(i in 1:dim(sim_awares)[1]){

cors[i] <- as.numeric(cor(sim_awares[i,], aware_true))

}

quantile(cors, probs = c(0.1, 0.5, 0.9))

# Previously it was

sim_awares <- extract(fit2, pars = 'aware')[[1]]

cors <- vector()

for(i in 1:dim(sim_awares)[1]){

cors[i] <- as.numeric(cor(sim_awares[i,], aware_true))

}

quantile(cors, probs = c(0.1, 0.5, 0.9))

# Repeat posterior checks, which show some additional improvements in terms of

# the realism of simulated data

pred_vacc <- extract(fit3, pars = 'vaccinate_preds')[[1]]

ppc_prev(pred_vacc, expert_boundaries = c(0, 0.6), variable = 'Rabies vaccination')

prob_vacc <- extract(fit3, pars = 'prob_vacc')[[1]]

ppc_prob(prob_vacc, variable = "vaccination against Rabies")

pred_aware <- extract(fit3, pars = 'aware')[[1]]

ppc_prob(pred_aware, variable = "'awareness' about Rabies", inv_logit = T)

# Now that our prior model is generating sensible data that is in-line with our

# domain expertise, we can condition the model on the vaccination outcomes

mod4 <- "data {

int<lower=0> N; // number of data points as an integer

int<lower=0,upper=1> vaccinate[N]; // binary outcomes

matrix[N, 3] edu; // education level is a matrix

int<lower=0> K; // number of knowledge questions

int<lower=0, upper=1> knowledge_answers[N,K]; // binary answers to knowledge questions

}

parameters {

vector[N] aware;

real alpha;

real<lower=0.5,upper=1> aware_sd;

real alpha_aware;

real beta_aware;

vector[K] alpha_questions;

vector[2] beta_edu_raw;

vector[2] beta_aware_edu_raw;

}

transformed parameters {

// Need first level of beta_edu coefs to be zero

vector[3] beta_edu;

vector[3] beta_aware_edu;

for (i in 2:3){

beta_edu[i] = beta_edu_raw[i-1];

beta_aware_edu[i] = beta_aware_edu_raw[i-1];

}

beta_edu[1] = 0;

beta_aware_edu[1] = 0;

}

model {

alpha ~ normal(-0.75, 1);

alpha_aware ~ normal(-0.5, 1);

beta_aware ~ normal(0.25, 1);

beta_edu_raw ~ normal(0.25, 1);

beta_aware_edu_raw ~ normal(0.25, 1);

aware_sd ~ uniform(0.5, 1);

// Latent awareness variable now depends on education

aware ~ normal(edu * beta_aware_edu, aware_sd);

// Model for question responses

alpha_questions ~ normal(-0.25, 1);

for (k in 1:K){

knowledge_answers[,k] ~ bernoulli_logit(to_vector(alpha_questions[k] + aware));

}

// Model for vaccination now included

vaccinate ~ bernoulli_logit(alpha + beta_aware * aware + edu * beta_edu);

}

generated quantities {

// Posterior predictions of vaccination

int<lower=0,upper=1> vaccinate_preds[N];

vector[N] prob_vacc;

prob_vacc = inv_logit(alpha + beta_aware * aware + edu * beta_edu);

vaccinate_preds = bernoulli_rng(prob_vacc);

// Posterior predictions for the knowledge questions

int<lower=0,upper=1> name_preds[N];

vector[N] prob_name;

prob_name = inv_logit(to_vector(alpha_questions[1] + aware));

name_preds = bernoulli_rng(prob_name);

int<lower=0,upper=1> trans_preds[N];

vector[N] prob_trans;

prob_trans = inv_logit(to_vector(alpha_questions[2] + aware));

trans_preds = bernoulli_rng(prob_trans);

}

"

# Condition the model on the full set of simulated data

fit4 <- stan(model_code = mod4,

data = model_data,

iter = 2000, chains = 4)

check_all_diagnostics(fit4)

# Check all estimated parameters against truths

stan_trace(fit4, 'alpha_questions')

stan_hist(fit4, 'alpha_questions')

summary(fit4, 'alpha_questions')$summary[,1:3]

name_alpha + alpha_aware_true; trans_alpha + alpha_aware_true

stan_hist(fit4, 'beta_edu')

summary(fit4, 'beta_edu')$summary[,1:3]

beta_edu_true

stan_hist(fit4, 'beta_aware_edu')

summary(fit4, 'beta_aware_edu')$summary[,1:3]

beta_aware_edu_true

# Our estimand of interest

stan_hist(fit4, 'beta_aware')

summary(fit4, 'beta_aware')$summary[,1:3]

beta_aware_true

# Check correlations among the simulated awareness and the truth

sim_awares <- extract(fit4, pars = 'aware')[[1]]

cors <- vector()

for(i in 1:dim(sim_awares)[1]){

cors[i] <- as.numeric(cor(sim_awares[i,], aware_true))

}

quantile(cors, probs = c(0.1, 0.5, 0.9))

# Previously it was

sim_awares <- extract(fit3, pars = 'aware')[[1]]

cors <- vector()

for(i in 1:dim(sim_awares)[1]){

cors[i] <- as.numeric(cor(sim_awares[i,], aware_true))

}

quantile(cors, probs = c(0.1, 0.5, 0.9))

#### Upgrading complexity to use an ordered knowledge question in addition to the two binary ones ####

# Simulating an ordered logit response host_true, representing knowledge of rabies hosts

# cutpoints will be 50th and 80th quantiles of the awareness variable

# https://mc-stan.org/docs/2_29/stan-users-guide/ordered-logistic.html

sim_ordinal = "

data {

int<lower=0> N; // number of data points as an integer

int<lower=1> K; // Number of ordinal response categories

vector[N] aware_true; // simulated awareness variable

vector[K-1] c; // internal cutpoints

}

generated quantities {

int<lower=1, upper=K> y[N]; // Simulated ordinal responses

for (n in 1:N)

y[n] = ordered_logistic_rng(aware_true[n], c);

}"

sim_ords <- stan(model_code = sim_ordinal, iter=1, chains=1,

algorithm="Fixed_param",

data = list(N = N,

aware_true = as.vector(aware_true),

# Three response categories

K = 3,

# Cutpoints are the 50th and 80th percentiles of the aware_true variable

c = as.vector(quantile(aware_true, probs = c(0.5, 0.8)))))

host_true <- as.vector(extract(sim_ords, 'y')[[1]])

plot(host_true, aware_true)

table(host_true)

# The model below avoids the non-identifiability of interior cut points in an ordered logit model

# by placing a principled prior model on the cutpoints that consistently regularizes them. This

# is particularly handy when we don't have much data from one or more of the ordered response categories

# https://betanalpha.github.io/assets/case_studies/ordinal_regression.html#1_Clear_Cut

mod5 <- "functions {

real induced_dirichlet_lpdf(vector c, vector alpha, real phi) {

int K = num_elements(c) + 1;

vector[K - 1] sigma = inv_logit(phi - c);

vector[K] p;

matrix[K, K] J = rep_matrix(0, K, K);

// Induced ordinal probabilities

p[1] = 1 - sigma[1];

for (k in 2:(K - 1))

p[k] = sigma[k - 1] - sigma[k];

p[K] = sigma[K - 1];

// Baseline column of Jacobian

for (k in 1:K) J[k, 1] = 1;

// Diagonal entries of Jacobian

for (k in 2:K) {

real rho = sigma[k - 1] * (1 - sigma[k - 1]);

J[k, k] = - rho;

J[k - 1, k] = rho;

}

return dirichlet_lpdf(p | alpha) + log_determinant(J);

}

}

data {

int<lower=0> N; // number of data points as an integer

int<lower=0,upper=1> vaccinate[N]; // binary outcomes

matrix[N, 3] edu; // education level is a matrix

int<lower=0> K; // number of knowledge questions

int<lower=0, upper=1> knowledge_answers[N,K]; // binary answers to knowledge questions

int<lower=1, upper=3> host_answers[N]; // answers about host of rabies

}

parameters {

vector[N] aware;

real alpha;

real alpha_aware;

real beta_aware;

real<lower=0.5,upper=1> aware_sd;

vector[K] alpha_questions;

vector[2] beta_edu_raw;

vector[2] beta_aware_edu_raw;

// cutpoints for ordered logistic model

ordered[2] c;

}

transformed parameters {

// Need first level of beta_edu coefs to be zero

vector[3] beta_edu;

vector[3] beta_aware_edu;

for (i in 2:3){

beta_edu[i] = beta_edu_raw[i-1];

beta_aware_edu[i] = beta_aware_edu_raw[i-1];

}

beta_edu[1] = 0;

beta_aware_edu[1] = 0;

}

model {

alpha ~ normal(-0.75, 1);

alpha_aware ~ normal(-0.5, 1);

beta_aware ~ normal(0.25, 1);

beta_edu_raw ~ normal(0.25, 1);

beta_aware_edu_raw ~ normal(0.25, 1);

aware_sd ~ uniform(0.5, 1);

// Latent awareness variable now depends on education

aware ~ normal(edu * beta_aware_edu, aware_sd);

// Model for question responses

alpha_questions ~ normal(-0.25, 1);

for (k in 1:K){

knowledge_answers[,k] ~ bernoulli_logit(to_vector(alpha_questions[k] + aware));

}

// Model for vaccination now included

vaccinate ~ bernoulli_logit(alpha + beta_aware * aware + edu * beta_edu);

// Model for response about rabies hosts, which uses the principled prior model

// on the internal cutpoints to regularise them toward sensible values when

// data is limited for one or more ordinal response categories

c ~ induced_dirichlet(rep_vector(1, 3), 0);

host_answers ~ ordered_logistic(aware, c);

}

generated quantities {

// Posterior predictions of vaccination

int<lower=0,upper=1> vaccinate_preds[N];

vector[N] prob_vacc;

prob_vacc = inv_logit(alpha + beta_aware * aware + edu * beta_edu);

vaccinate_preds = bernoulli_rng(prob_vacc);

// Posterior predictions for the knowledge questions

int<lower=0,upper=1> name_preds[N];

vector[N] prob_name;

prob_name = inv_logit(to_vector(alpha_questions[1] + aware));

name_preds = bernoulli_rng(prob_name);

int<lower=0,upper=1> trans_preds[N];

vector[N] prob_trans;

prob_trans = inv_logit(to_vector(alpha_questions[2] + aware));

trans_preds = bernoulli_rng(prob_trans);

}

"

# Condition the model on the full set of simulated data

model_data$host_answers <- host_true

fit5 <- stan(model_code = mod5,

data = model_data,

iter = 2000, chains = 4)

check_all_diagnostics(fit5)

# Our previous correlation between estimated awareness and the truth was:

sim_awares <- extract(fit4, pars = 'aware')[[1]]

cors <- vector()

for(i in 1:dim(sim_awares)[1]){

cors[i] <- as.numeric(cor(sim_awares[i,], aware_true))

}

quantile(cors, probs = c(0.1, 0.5, 0.9))

# And now it has been majorly improved:

sim_awares <- extract(fit5, pars = 'aware')[[1]]

cors <- vector()

for(i in 1:dim(sim_awares)[1]){

cors[i] <- as.numeric(cor(sim_awares[i,], aware_true))

}

quantile(cors, probs = c(0.1, 0.5, 0.9))

# How is the precision of our estimate for the estimand of interest?

stan_hist(fit5, 'beta_aware')

summary(fit5, 'beta_aware')$summary[,1:3]

beta_aware_true

# Previously it was:

summary(fit4, 'beta_aware')$summary[,1:3]

# Other estimated coefficients

stan_hist(fit5, 'beta_edu')

beta_edu_true

stan_hist(fit5, 'beta_aware_edu')

beta_aware_edu_true

# How does this model distinguish between vaccinated and unvaccinated dogs?

layout(matrix(1:2, nrow=2, ncol=1))

hist(extract(fit5, 'prob_vacc')[[1]][,vaccinate_true==0],

main = 'model 5', xlab = '', ylab = '', freq = F,

col = 'darkgrey', xlim = c(0, 1))

hist(extract(fit5, 'prob_vacc')[[1]][,vaccinate_true==1],

add = T, freq = F, col = "#A2505050",

border = "#7C000070", xlim = c(0, 1))

# How did the previous model compare?

hist(extract(fit4, 'prob_vacc')[[1]][,vaccinate_true==0],

main = 'model 4', xlab = '', ylab = '', freq = F,

col = 'darkgrey', xlim = c(0, 1))

hist(extract(fit4, 'prob_vacc')[[1]][,vaccinate_true==1],

add = T, freq = F, col = "#A2505050",

border = "#7C000070", xlim = c(0, 1))

layout(1)

# A function to compute and summarise posterior Brier scores against the true observed

# vaccination outcome

summarise_brier = function(truth, predictions){

brier_scores = vector(length = length(truth))

for(i in 1:length(brier_scores)){

brier_scores[i] = mean((predictions[,i] - truth[i])^2, na.rm = TRUE)

}

brier_scores

}

briers5 <- summarise_brier(truth = vaccinate_true,

predictions = extract(fit5, 'prob_vacc')[[1]])

# Look for any systematic variation in predictive calibration; here we ask if predictive scores are

# worse (larger values) among different vaccination states or levels of education, but the same principle could be used to

# look for patterns over space, or over respondent ages etc... to examine whether there is any remaining

# structure in the data the the model is failing to replicate

# In general the predictions are better calibrated when predicting the true 0s (unvaccinated) than

# when predicting the true 1s (vaccinated)

hist(briers5[vaccinate_true==0])

hist(briers5[vaccinate_true==1])

confint(lm(briers5 ~ vaccinate_true))

# This translates into worse scores at higher levels of education due to the fact that education is positively

# predictive of vaccination

confint(lm(briers5 ~ edu_true_factor))
