# Supplementary material for: Factors influencing canine rabies vaccination among dog-owning households in Nigeria
Source: One Health. 2024 May 10;18:100751. doi: 10.1016/j.onehlt.2024.100751 (PMC11141449; doi:10.1016/j.onehlt.2024.100751)
Supplement: Supplementary file 3 — Supplementary material 3 [file mmc3.docx]

**Table 2**: **Posterior mean, odd ratio, standard deviation, credible intervals, and convergence diagnostics for estimated effects on the logit (probability of vaccination) among dog owners in Nigeria**

| **Characteristic** | Mean | OR, Crl(2.5%- 97.5%) | sd | 75% | 97.5% Crl | Rhat |
| --- | --- | --- | --- | --- | --- | --- |
| **Occupation** | | | | | | |
| Civil Servant | 0.584 | 1.79 (1.44-2.26) | 0.116 | 0.663 | 0.813 | 1 |
| Private Sector | -0.211 | 0.81(0.69-0.96) | 0.085 | -0.152 | -0.045 | 1 |
| **What treatment option would you opt for in the event of a dog bite?** | | | | | | |
| **Reference** (Contemporary medicine) | | | | | | |
| Traditional treatment | -0.831 | 0.44 (0.46-0.52) | 0.091 | -0.768 | -0.653 | 1 |
| **Number of dogs in the household** | | | | | | |
| **Reference** (one dog) | | | | | | |
| More than one | -0.147 | 0.86 (0.73-1.02) | 0.086 | -0.089 | 0.024 | 1 |
| **Location of the dog owner** | | | | | | |
| Anambra and Enugu | -0.543 | 0.58 (0.47-0.73) | 0.116 | -0.464 | -0.314 | 1 |
| FCT | 0.585 | 1.80 (1.42-2.29) | 0.123 | 0.669 | 0.829 | 1 |
| **Feeding** | | | | | | |
| The dog is on its own | 0.190 | 1.21 (0.97-1.53) | 0.117 | 0.270 | 0.424 | 1 |
| Cook special meal | 0.874 | 2.40 (1.90-3.07) | 0.121 | 0.954 | 1.122 | 1 |
| **Dog use** | | | | | | |
| Pet | 0.259 | 1.30 (1.04-1.62) | 0.116 | 0.338 | 0.485 | 1 |
| Breeding and hunting | 0.277 | 1.32 (1.08-1.62) | 0.103 | 0.348 | 0.481 | 1 |
| **Is your dog under confinement?** | | | | | | |
| No | -0.427 | 0.65 (0.53-0.81) | 0.111 | -0.353 | -0.208 | 1 |
| Yes | 0.931 | 2.54 (2.08-3.10) | 0.103 | 0.999 | 1.130 | 1 |
| **Awareness** | | | | | | |
| Latent awareness | 0.898 | 2.46 (2.62-3.01) | 0.099 | 0.964 | 1.102 | 1 |
